# Positive feedback loop of IL-1β/Akt/RARα/Akt signaling mediates oncogenic property of RARα in gastric carcinoma

**DOI:** 10.18632/oncotarget.14267

**Published:** 2016-12-27

**Authors:** Hong-Yue Ren, Fan Liu, Gui-Li Huang, Yu Liu, Jin-Xing Shen, Pan Zhou, Wen-Ming Liu, Dong-Yan Shen

**Affiliations:** ^1^ Department of Pathology, The Affiliated Southeast Hospital of Xiamen University, Zhangzhou 363000, Fujian Province, China; ^2^ Department of Medical College, Xiamen University, Xiamen 361005, Fujian Province, China; ^3^ State Key Laboratory of Cellular Stress Biology, Key Laboratory of the Ministry of Education for Coastal and Wetland Ecosystems, Department of Life Sciences, Xiamen University, Xiamen 361005, Fujian Province, China; ^4^ Department of Biobank, The First Affiliated Hospital of Xiamen University, Xiamen 361003, Fujian Province, China; ^5^ Department of Gastroenterology, Zhongshan Hospital, Gastroenterology Institute of Xiamen University, Gastroenterology Center of Xiamen, Xiamen 361003, Fujian Province, China

**Keywords:** RARα, gastric carcinoma, Akt, IL-1β

## Abstract

Abnormal expression and function of retinoic acid receptor α (RARα) have been reported to be associated with various cancers including acute promyelocytic leukemia and hepatocellular carcinoma. However, the role and the mechanism of RARα in gastric carcinoma (GC) were unknown. Here, the expression of RARα was frequently elevated in human GC tissues and cell lines, and its overexpression was closely correlated with tumor size, lymph node metastasis and clinical stages in GC patients. Moreover, RARα overexpression was related with pathological differentiation. Functionally, RARα knockdown inhibited the proliferation and metastasis of GC cells, as well as enhanced drug susceptibility both *in vitro* and *in vivo*. Additionally, RARα knockdown suppressed GC progression through regulating the expression of cell proliferation, cell cycle, invasion and drug resistance associated proteins, such as PCNA, CyclinB1, CyclinD2, CyclinE, p21, MMP9 and MDR1. Mechanistically, the above oncogenic properties of RARα in GC were closely associated with Akt signaling activation. Moreover, overexpression of RARα was induced by IL-1β/Akt signaling activation, which suggested a positive feedback loop of IL-1β/Akt/RARα/Akt signaling in GC. Taken together, we demonstrated that RARα was frequently elevated in GC and exerted oncogenic properties. It might be a potential molecular target for GC treatment.

## INTRODUCTION

Gastric carcinoma (GC) is ranked as the second most common cancer and is the leading cause of cancer-related deaths in the world [1]. Current available therapeutic methods for GC, such as surgical excision, chemotherapy and chemoembolization, are less optimal especially in late stage of patients [2]. Therefore, it is urgent to understand the molecular mechanisms underlying GC progression so as to develop novel molecular targets for more effective therapies.

Retinoids including vitamin A and retinoic acid derivatives have profound effect on embryogenesis, differentiation, and carcinogenesis [3, 4]. These biological activities of retinoids are mediated by retinoic acid receptors (RARs) and retinoid X receptors (RXRs) two classes of specific nuclear retinoid receptors in the steroid/thyroid hormone family. RAR consist of three distinct receptor subtypes: α, β, and γ [5, 6]. Among them, increasing lines of evidence have implied that aberrant expression of RARα may play critical roles in carcinogenesis. For example, in acute promyelocytic leukemia patients, the RARα gene is fused with a number of alternative partner genes such as PML, promyelocytic leukemia zinc finger [7] and nucleophosmin [8]. RARα has been identified as highly expressed in hepatocellular carcinoma (HCC), and is possibly responsible for abnormal growth of HCC [9]. However, the role and mechanism of RARα in GC remain unknown.

It is widely recognized that infection with Helicobacter pylori plays a crucial role in the initiation and progression of GC [10]. In recent years, the significance of chronic inflammation in carcinogenesis has gained more and more attention [11]. Such inflammatory response seems to be regulated by many proinflammatory cytokines, that can have autocrine, paracrine and endocrine effects. Interlenkin-1β (IL-1β) is a member of proinflammatory cytokine family which are produced by both tumor cells and stromal cells [12]. It has been reported to govern the proliferation and invasion related proteins of GC [13, 14]. Nevertheless, whether IL-1β regulates RARα expression in development and progression of GC is unclear.

Cytokine and other growth factors such as insulin, epidermal growth factor and vascular endothelial growth factor can trigger the activation of signaling pathway including phosphatidylinositide 3-kinase (PI3K)/Akt through binding to their cell surface receptors. Moreover, PI3K/Akt signaling pathway regulates a variety of cellular activities, in particular, previous reports have indicated it has participated in tumorigenesis and tumor progression by promoting cell proliferation [15, 16]. In GC, phosphorylated Akt (p-Akt) expression may not only be useful for predicting the prognosis and efficacy of fluorouracil treatment [17], and also reflect the grade of malignancy in human gastric adenocarcinomas [18]. PI3K/Akt signaling is required for the attachment and spreading and growth *in vivo* of metastatic scirrhous GC as well [19]. However, whether the regulation of PI3K/Akt signaling links RARα and GC remains undefined.

In the present study, we found that the expression of RARα was highly expressed in both GC patients and cell lines. Moreover, RARα level is significantly related with the progression of GC. Knockdown of RARα could suppress PI3K/Akt signaling, leading to inhibition metastatic abilities and increasing drug susceptibility of GC cells. Futhermore, the proinflammation cytokine IL-1β could induce overexpression of RARα through activation of PI3K/Akt signaling. Thus, we identify a positive feedback loop of IL-1β/Akt/RARα/Akt signaling, suggesting an oncogenic potential of RARα involved in the development of GC.

## RESULTS

### Overexpression of RARα and its prognostic values in GC patients

**Figure 1:**
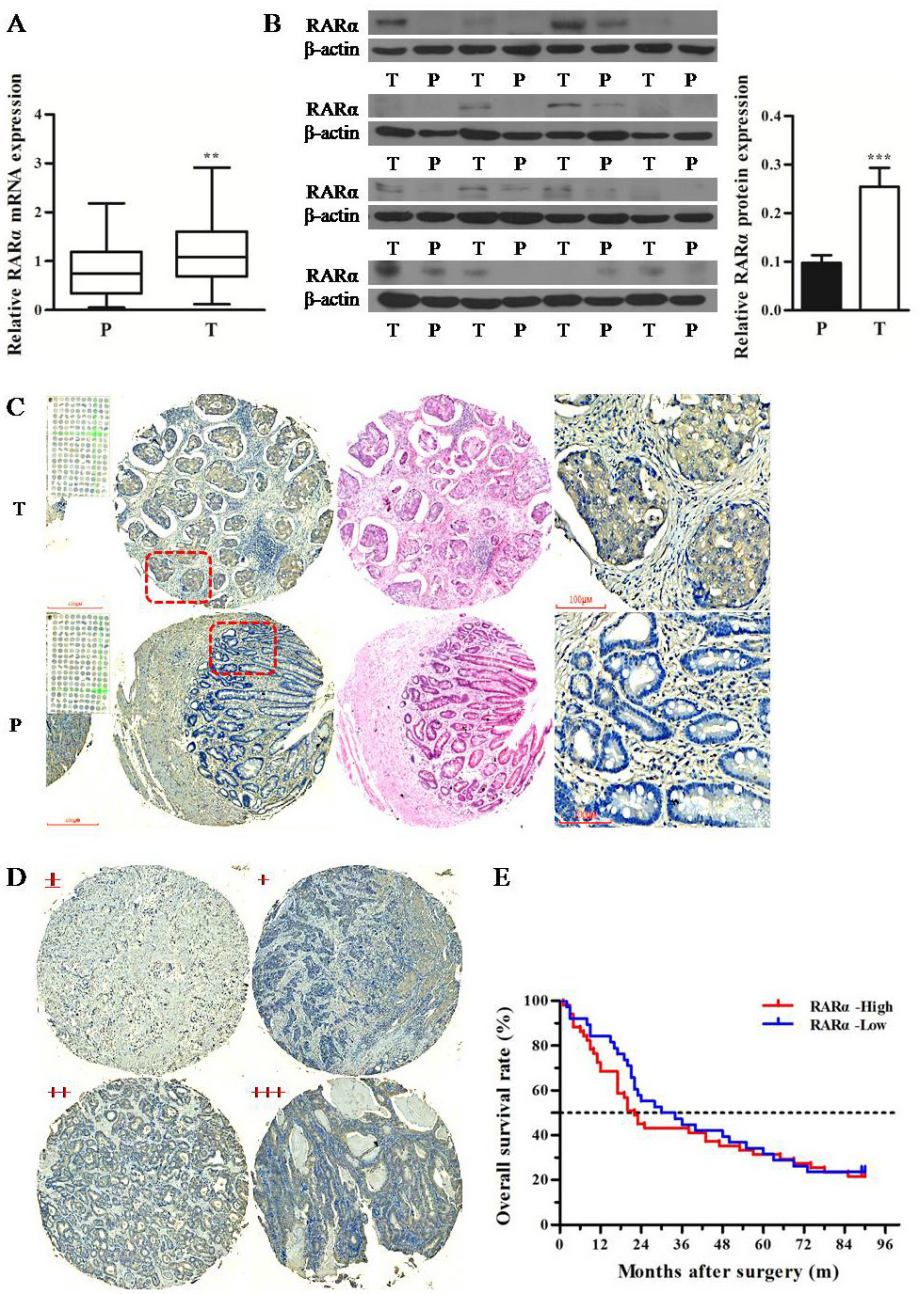
Expression and clinical significance of RARα in GC tissues **A**. Expression of RARα mRNA in T and P tissues was assessed by Q-PCR. The data were normalized to GAPDH. The error bars presented as mean ± SEM. **B**. Expression of RARα protein in T and P tissues was assessed by Western blot. β-actin served as loading control. **C**. IHC analysis of RARα protein in GC tissue microarray. Zoom: 100×, 400×. **D**. Four grades of RARα staining intensity. **E**. Kaplan–Meier survival analysis of RARα expression in GC patients. T, tumor; P, paracarcinoma tissues; ** *P* < 0.01, *** *P* < 0.001.

Q-PCR and Western blot were performed to detect the expression of RARα in tumor (T) and paired paracarcinoma (P) tissues of 21 GC patients. The results revealed that the mRNA and protein levels of RARα were significantly elevated in T tissues compared with that in P tissues (Figure 1A-1B). More samples from a cohort of 180 GC patients in tissue microarray were subjected to evaluate the expression and clinical significance of RARα in GC. The IHC results showed that RARα protein was strongly expressed in T tissues, presented dominantly in cytoplasm of tumor cells, but was weakly or not stained in P tissues (Figure 1C). In order to investigate the level of RARα in these two tissues, the staining intensity of RARα was divided into low (- to +) or high (++ to +++) groups (Figure 1D). As shown in Table 1, the high expression rate of RARα in T tissues was 58%, which was much higher compared with the 6.25% in P tissues.

**Table 1:** Distribution of RARα classifications in GC and paracarcinoma tissues

Tissue type	n	RARα stain grades				*X^2^*	*P*
		-	+	++	+++		
Tumor	100	29	13	29	29	56.25	< 0.0001
Paracarcinoma	80	40	35	5	0		

Clinicopathological analysis revealed that overexpression of RARα was strongly correlated with tumor diameter, lymph node metastasis and clinical stages in GC patients (Table 2). Meanwhile RARα overexpression was related with pathological differentiation. Furthermore, Kaplan-Meier analysis showed that GC patients with high RARα expression had shorter median survival time (22 months) than that of low RARα expression patients (32 months), but there was no statistical significance between the two groups (*P* > 0.05) (Figure 1E). Taken together, these results indicated that abnormal overexpression of RARα might be associated with the development of GC.

**Table 2:** Relationships between RARα and clinical features in GC

Features	n	RARα		*X^2^*	*P*
		Low	High		
Age				1.301	0.254
< 60	33	16	17		
≥ 60	67	26	41		
Sex				0.016	0.8986
female	35	15	20		
male	65	27	38		
Pathological diferentiation				16.980	0.0007*
II	15	8	7		
II-III	22	4	18		
III	52	20	32		
III-IV	11	10	1		
Tumor diameter				9.514	0.0086*
d ≤ 4	29	19	10		
4 < d < 10	58	18	40		
d ≥ 10	13	5	8		
T classification				6.004	0.1114
T1	9	7	2		
T2	11	5	6		
T3	66	26	40		
T4	14	4	10		
N classification				9.549	0.0228*
N0	27	18	9		
N1	16	5	11		
N2	27	10	17		
N3	30	9	21		
M classification				0.305	0.5808
M0	91	39	52		
M1	9	3	6		
Clinical stages				13.87	0.0031*
1	12	9	3		
2	32	18	14		
3	49	14	35		
4	7	1	6		

* A *P* value of less than 0.05 was considered statistically significant.

### Construction of RARα knockdown cell model in GC cell lines

**Figure 2:**
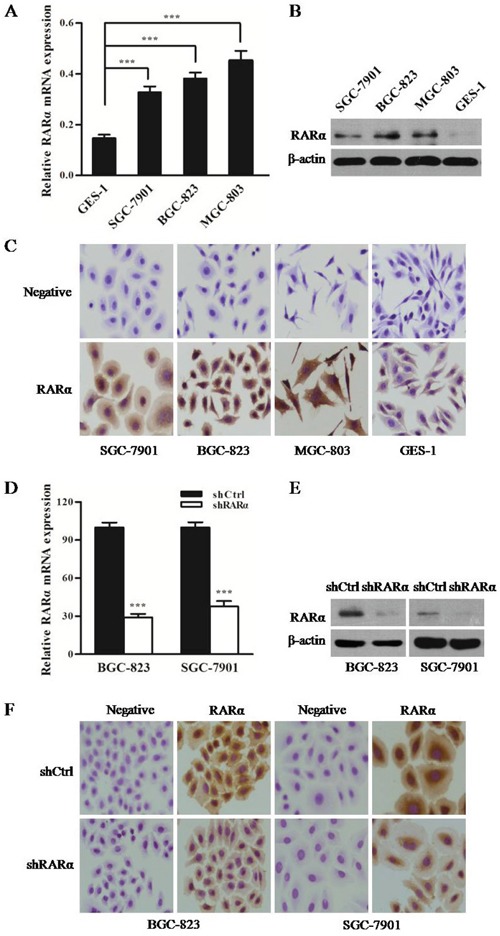
Expression of RARα in GC cell lines and constructed cell models **A**. Expression of RARα mRNA in GC cells was detected by Q-PCR. The data were normalized to GAPDH. The error bars presented as mean ± SEM. **B**. Expression of RARα protein in GC cells was detected by Western blot. **C**. ICC analysis of RARα in GC cells. Zoom: 200×. **D-F**. Construction of RARα knockdown cell model in SGC-823 and SGC-7901 were identified by Q-PCR, Western blot and ICC. *** *P* < 0.001.

To further determine the aberrant expression pattern of RARα in GC, three GC cell lines (BGC-823, SGC-7901, and MGC-803) and a normal human gastric epithelial cell line GES-1were subjected to detect the level of RARα. As shown in Figure 2A-2B, the mRNA and protein levels of RARα in three GC cell lines were markedly higher than those of GES-1 cell lines. Moreover, overexpression of RARα was primarily localized in the cytoplasm of GC cells, that was consistent with the IHC results from clinical GC tissues (Figure 2C). In order to characterize the functional role of RARα in the development of GC, RARα was downregulated by stably transducing with shRARα-expressing lentiviruses. As shown in Figure 2D-2F, the mRNA and protein levels of RARα in shRARα-BGC-823 or SGC-7901 cells were much lower than those in their respective control cells, indicating that RARα was effectively knockdown.

### Inhibitory effects of RARα knockdown on proliferation of GC cells

**Figure 3:**
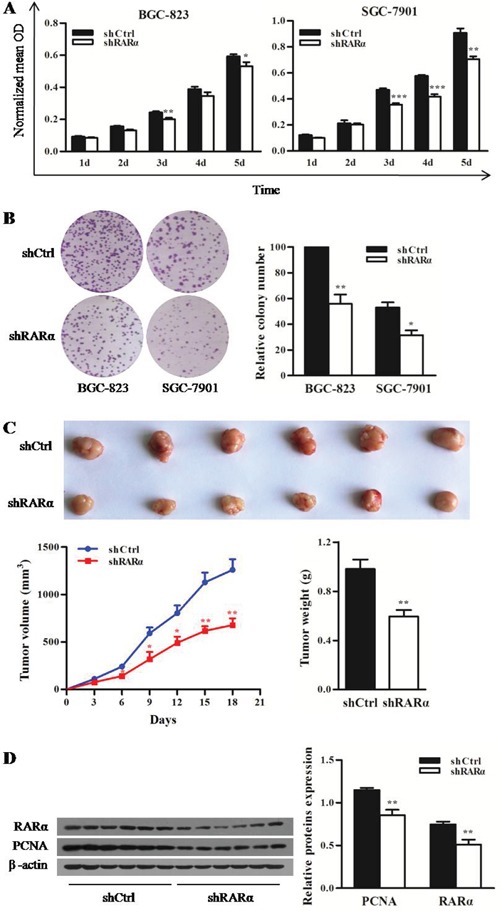
Effect of RARα knockdown on the proliferation of GC cells in vitro and in vivo **A**. MTT analysis for the proliferation of BGC-823 and SGC-7901 cells after RARα knockdown. **B**. Control or RARα knockdown of BGC-823 and SGC-7901 cells were subjected to anchorage-dependent clonogenic assay. Representative pictures for colony growth are shown (right). Quantification of the number of colonies was normalized with that of shCtrl-BGC-823 cells (left). **C**. shRARα-BGC-823 cells and control cells were subcutaneously injected in nude mice to establish xenograft tumors. Representative tumors and their volume are depicted graphically; n = 6 (for each group). Error bar represents SEM. **D**. Western blot analysis for PCNA and RARα in shCtrl and shRARα xenograft tumors with β-actin as a loading control. * *P* < 0.05, ** *P* < 0.01. *** *P* < 0.001.

To characterize the biological effect of RARα knockdown on the proliferation of GC cells *in vitro*, MTT and colony formation assay were performed. Compared with the control cells (Figure 3A), the proliferation of both shRARα-BGC-823 and shRARα-SGC-7901 cells were significantly reduced from day 3. Moreover, colonies of shRARα-BGC-823 and shRARα-SGC-7901 cells were fewer and relatively smaller in size, compared with their relative shCtrl cells (Figure 3B).

The tumorigenic potential of RARα was also evaluated by xenograft tumor in nude mice. As shown in Figure 3C, the growth of shRARα-BGC-823 xenografts were slower from day 6 compared with that of shCtrl group. The average tumor weight of shRARα-BGC-823 xenografts was 0.59 ± 0.05 g, which was significantly lower than that of shCtrl group (0.98 ± 0.08 g). In addition, significant reduction of proliferation marker PCNA was observed in shRARα-BGC-823 xenografts (Figure 3D). Taken together, these results suggested that RARα might be critical for oncogenic property of GC cells.

### RARα functions in cellular motility, invasion and drug susceptibility

**Figure 4:**
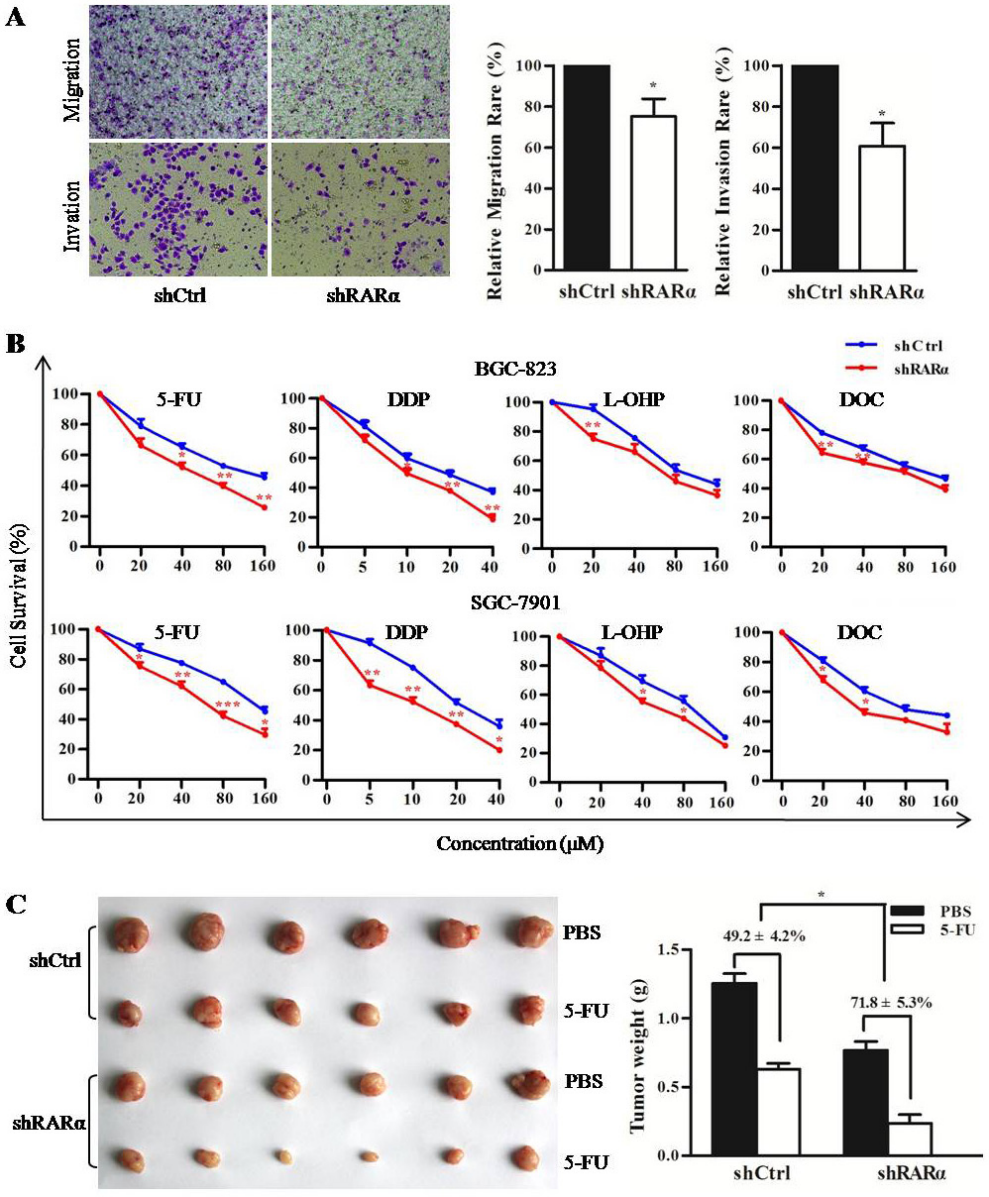
Critical role of RARα in cellular motility, invasion and drug susceptibility **A**. Control or RARα knockdown of BGC-823 were subjected to transwell migration (without Matrigel) and invasion (with Matrigel) assays. Cells invading Matrigel were imaged in a bright-field microscope under 200× magnification (right). Image software analysis for the results of three independent experiments (left). **B**. MTT assay performed in shCtrl or shRARα of BGC-823 and SGC-7901 to detected the drug susceptibility to 5-FU, DDP, L-OHP and DOC. **C**. The drug susceptibility of shCtrl or shRARα of BGC-823 xenograft tumor to 5-FU were shown (right) and analyzed (left), n = 6 (for each group). * *P* < 0.05, ** *P* < 0.01, *** *P* < 0.001.

Metastasis is not only a sign of deterioration but also a major cause of treatment failure in GC patients. Hence transwell assays were performed to detect the effect of RARα knockdown on the capacity of migration and invasion in BGC-823 cell line. Transwell assays results showed a significant reduction in migration of shRARα cells compared to that of shCtrl cells in BGC-823 cell line. To further explore the role of RARα in cell invasion, transwell assays with matrigel precoated in the chamber were performed and demonstrated that the invasive capacity of BGC-823 cells was markedly reduced after RARα knockdown (Figure 4A).

Multidrug resistance is frequently induced during chemotherapy. The effect of RARα knockdown on the drug susceptibility of GC cells was investigated *in vitro* and *in vivo*. As shown in Figure 4B, MTT assays revealed a significant enhancement in several chemotherapeutics susceptibility of shRARα cells compared to shCtrl cells in both BGC-823 and SGC-7901 cells. Furthermore, the growth inhibition rate of 5-FU on RARα knockdown xenograft tumors (71.8 ± 5.3%) was much higher than that of control group (49.2 ± 4.2%) (Figure 4C). These above data indicated that RARα knockdown could inhibit metastatic abilities and enhance drug susceptibility of GC cells.

### RARα promotes cell proliferation, invasion and inhibits drug susceptibility via activation of PI3K/Akt signaling

**Figure 5:**
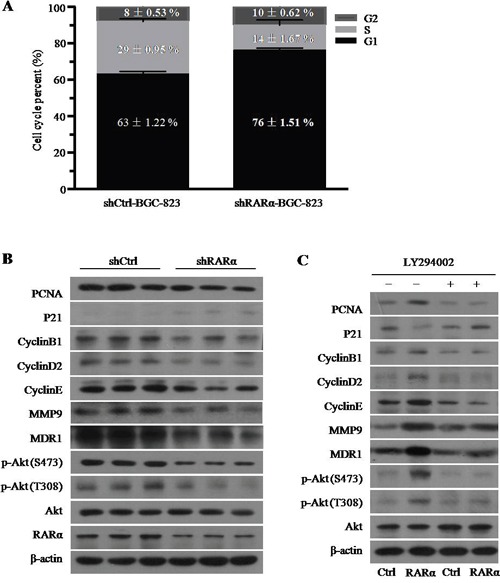
The role and mechanism of RARα in the GC cell development and progression **A**. Cell cycle was analysed by flow cytometry after RARα knockdown. **B**. Western blot analysis of cell cycle, proliferation, invasion, drug resistance and Akt signaling associated proteins expression after RARα knockdown in BGC-823. **C**. Effect of LY294002 and (or) RARα overexpression on the expression of cell cycle, proliferation, invasion, drug resistance and Akt signaling associated proteins expression in BGC-823. LY294002, 1μM.

Cell cycle disorder is closely related to the rapid proliferation of tumor cells. The flow cytometry data of cell cycle analysis showed that significant increase in the proportion of cells in the G1 phase were detected in RARα knockdown, and accordingly, the fractions of cells in the S phase decreased (Figure 5A and Supplementary Figure 1). Western blot analysis showed that after silencing of RARα, the expression of PCNA, CyclinB1, CyclinD2, and CyclinE was decreased, whereas p21 expression was augmented compared to the control (Figure 5B). In addition, MMP9 and MDR1 have been recognized as critical invasion and drug resistance makers, respectively. As shown in Figure 5B, the expression levels of MMP9 and MDR1 were inhibited after downregulation of RARα.

RARα could interact with p85α of PI3K subunits to activate PI3K/Akt signaling [20], which played an important role in the development of GC including regulation of cell cycle [21]. Consistent with previous studies, we found that knockdown of RARα suppressed the phosphorylation level of Akt at serine 473 (S473) and threonine 308 (T308) (Figure 5B). In contrast, overexpression of RARα strongly increased the level of p-Akt at S473 and T308 and this effect could be retarded by LY294002, the specific inhibitor for PI3K/Akt signaling (Figure 5C). Meanwhile, LY294002 could reverse the effect of RARα overexpression on the expression of PCNA, CyclinB1, CyclinD2, CyclinE, p21, MMP9 and MDR1 (Figure 5C), which indicated that RARα might regulate the cell development and progression via activation of PI3K/Akt signaling.

### A positive feedback loop between RARα and the Akt signaling

**Figure 6:**
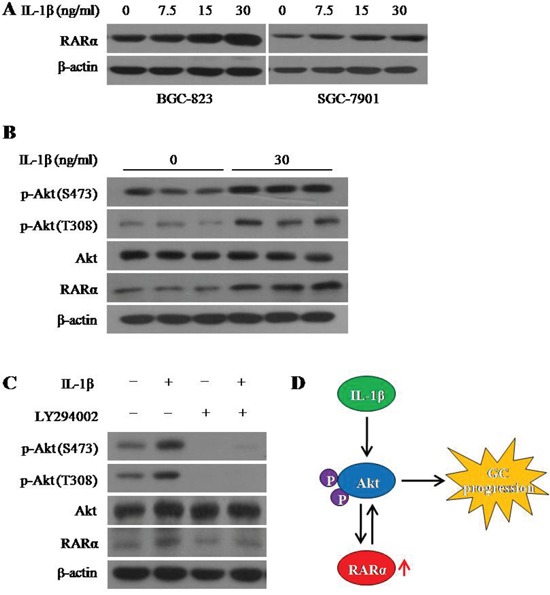
IL-1β induced RARα overexpression via activation Akt signaling **A**. Western blot analysis for RARα expression after treatment with different concentration of IL-1β for 24 hour. **B**. Western blot analysis for RARα expression and the activation of Akt signaling after treatment with IL-1β for 24 hour in BGC-823. **C**. After treatment with IL-1β and (or) LY294002 for 24 hour, expression of RARα, p-Akt and Akt were detected by Western blot in BGC-823. IL-1β, 30 ng/ml; LY294002, 1 μM. **D**. Schematic diagram for the positive feedback loop of IL-1β/Akt/RARα/Akt signaling.

In order to investigate the molecular mechanisms underlying the RARα aberrant expression in GC, we hypothesized that the infiltration of inflammatory factors, an important feature of the tumor microenvironment, lead to the overexpression of RARα. During screening experiments, we found that IL-1β, an important proinflammatory cytokine, increased the level of RARα protein in a dose-dependent manner in both BGC-823 and SGC-7901 cells (Figure 6A), whereas TNFα and IL-6 failed to alter RARα expression significantly in GC cells (Supplementary Figure 2). Furthermore, IL-1β activated the PI3K/Akt signaling via enhancing the phosphorylation of Akt at S473 and T308 (Figure 6B). Meanwhile, overexpression of RARα induced by IL-1β could be reversed by the PI3K/Akt inhibitor LY294002 (Figure 6C), indicating that Akt signaling is essential for IL-1β-upregulated RARα. Hence, these data suggested the existence of a positive feedback loop of IL-1β/Akt/RARα/Akt signaling in the development of GC (Figure 6D).

## DISCUSSION

Multiple lines of evidence presented here identified an aberrant expression of RARα in providing oncogenic cues that promote GC tumorigenesis. We not only validated the frequent upregulation of RARα in clinical GC specimens and its clinical significance, and also demonstrated the oncogenic property of RARα in proliferation, metastasis and drug susceptibility of GC cells through Akt signaling. Our results suggested that targeting RARα might be a potentially beneficial for treatment of GC.

RARα has been characterized as an oncogene in the development of various tumors. Its overexpression disrupted normal acinar structure and induced epithelial to mesenchymal transition during mammary tumorigenesis [22]. RARα is also required for efficient estrogen receptor α mediated transcription and cell proliferation in breast cancer [23]. Our current study showed that, downregulation of RARα inhibited GC growth and increased drug susceptibility both *in vitro* and *in vivo*, as well as decreased metastasis *in vitro*. RARα knockdown resulted in cell cycle arrest at G1 phase accompanying with the reduction of S phase. In addition, silencing RARα inhibited the expression of PCNA, CyclinB1, CyclinD2, CyclinE, MMP9 and MDR1 and enhanced p21 expression, that may play critical roles in tumor growth, invasion, drug resistance. All results came to a conclusion that RARα regulate the development and progression of GC in a form of oncogene.

Molecular studies provided evidences that, RARs including RARα could interact with p85α in various cell types thus activate the PI3K/Akt signaling pathway [24, 25]. PI3Ks can be divided into three classes according to structure and function, termed as class I, II and III. Class IA PI3Ks consists of regulatory and catalytic subunits. P85α, a regulatory subunit of class IA PI3Ks, appears to be a phosphoprotein substrate of many cytoplasmic and receptor tyrosine kinases [26]. We further evaluated the biological significance of the regulation between RARα and PI3K/Akt signaling in the growth of GC cells. Our data showed that the phosphorylation of Akt was suppressed by downregulation of RARα, and enhanced by upregulation of RARα. Moreover, RARα overexpression increased the level of PCNA, CyclinB1, CyclinD2, CyclinE MMP9 and MDR1 as well as decreased p21 expression. And this could be reversed by LY294002, the specific inhibitor for PI3K/Akt signaling, suggesting that RARα might regulate PI3K/Akt signaling to promote the progression of GC.

In the present study, we showed that RARα localized majorly in the cytoplasm of clinical samples and cell lines, which was consistent with RARγ. Previous studies have shown that overexpressed RARγ could translocate to the cytoplasm in HCC and cholangiocarcinoma [27, 28]. Altered subcellular localization of RARγ was associated with its oncogenic effects. In addition, RARγ could interact with p85α and β-catenin, that may retain RARγ in the cytoplasm [27, 28].

The cause and mechanism of abnormal RARα overexpression in malignant tumors have not yet been studied. Inflammation of stomach mucosa has been regarded as a key cause of gastric carcinogenesis, and the presence of proinflammatory cytokines can regulate specific genes participated in the process of GC. IL-1β, serving as a known proinflammatory cytokine, can activate MUC2 gene involved in the gastric neoplassic transformation [29]. Some studies indicated that IL-1β can trigger the activation of both Akt pathway and NF-κB pathway, which appears to be important molecular links between inflammation and tumor [30, 31]. In the present study, we found that IL-1β and not TNFα or IL-6 exerted potent effects on the expression of RARα. IL-1β could enhance RARα overexpression in a dose-dependent manner. In addition, after co-incubation with IL-1β, the phosphorylation of Akt was enhanced. LY294002 inhibited the upregulation of RARα induced by IL-1β, suggesting that IL-1β upregulated RARα via Akt signaling. It is likely that overexpression of RARα promoted GC progression via Akt signaling and this further formed a positive feedback of IL-1β/Akt/RARα/Akt signaling.

In conclusion, our study identified RARα as a potential oncogene in the progression of GC. Overexpression of RARα might promote the progression of GC cells through forming the positive feedback of IL-1β/Akt/RARα/Akt signaling. Therefore, our findings are potentially beneficial for the future development of promising target against malignant progression of GC.

## MATERIALS AND METHODS

### Reagents

1640 medium, DMEM medium, fetal bovine serum (FBS), penicillin and Lipofectamine 2000 were purchased from Invitrogen (Carlsbad, CA, USA). IL-1β was from R&D Systems. LY294002, propidium iodide (PI), thymidine, 5-fluorouracil (5-FU), diaminodichloroplatinum (DDP), oxaliplatin (L-OHP) and docetaxel (DOC) were from Sigma-Aldrich (Indianapolis, IN, USA). Antibodies against RARα (sc-551), PCNA (sc-25289), CyclinB1 (sc-752), CyclinD2 (sc-181), CyclinE (sc-481) and MMP9 (sc-21733) were from Santa Cruz Biotechnology (Santa Cruz, CA, USA). Antibodies against β-actin (12262), p-Akt (Ser473, 12694), p-Akt (Thr308, 13038), Akt (4685) and MDR1 (12683) were from Cell Signaling Technologies (Danvers, Ma, USA). The EliVision Plus kit and Quantitative PCR kit were from Maixin Bio (Fuzhou, China) and Tiangen Bio (Beijing, China), respectively.

### Patients and tissue samples

Paired tumor (T) and paracarcinoma tissues (P) (> 5 cm distant from the edges of tumor; T = P, n = 21) specimens of GC patients, who had undergone a gastrectomy between 2013 and 2015, were collected at the First Affiliated Hospital of Xiamen University. Tissue microarray of human GC was purchased from National SOBC Biobank, which included 100 GC specimens, 80 samples of paired paracarcinoma tissues. The clinicopathological data are summarized in Table 2 including age, sex, pathological diferentiation, tumor diameter, TNM classifications and Clinical stages. None of patients received preoperative treatment, such as radiation or chemotherapy. Written informed consent was obtained from each patient prior to sample collection. The study protocol was conducted in accordance with the ethical guidelines of the 1975 Declaration of Helsinki and was approved by the Institute Research Ethics Committee of the First Affiliated Hospital of Xiamen University.

### Cell culture and transfection

Human GC cell lines BGC-823, SGC-7901, and MGC-803 as well as normal human gastric epithelial cells GES-1 were supplied by Key Laboratory of the First Affiliated Hospital of Xiamen University. The cells cultured in RPMI 1640 medium containing 10% FBS, 100 U/ml penicillin at 37°C in an atmosphere of 5% CO^2^ and humidified incubator. For establishment of RARα knockdown cell lines, 293T cells were cotransfected with target plasmids and the packaging plasmids according to the manufacturer's instructions. Viral supernatants including infectious lentiviral particles were harvested after transfection day 2, and then added in BGC-823 and SGC-7901culture. After infection, cells were selected in 500 μg/ml G418 for 2 weeks.

### Immunohistochemistry (IHC) and immunocytochemistry (ICC)

IHC and ICC were performed as previously described [32]. Tissue microarray and slides of cells were reacted with primary antibody to RARα (1:100) at 4°C for overnight. Pre-immune sheep serum was used as negative control. All sections were independently and blindly examined by two experienced pathologists.

### Quantitative real-time reverse transcription PCR assay (Q-PCR)

The Q-PCR was performed on an ABI 7500 fast real-time PCR system (Applied Biosystems, Foster City, CA) as previously described [33]. The Q-PCR primers are as follows: RARα, forward primer AATACACTACGAACAACAGC, reverse primer CGAACTCCACAGTCTTAATG; GAPDH, forward primer CACATGGCCTCCAAGGAGTAAG, reverse primer TGAGGGTCTCTCTCTTCCTCTTGT. Relative expression levels of RARα were normalized against GAPDH.

### Western blot analysis

Cell lysates were separated by SDS–PAGE and transferred to a PVDF membrane as previously described [33]. After incubation with primary and secondary antibodies, the membranes were washed in PBST. Then signals were detected with an enhanced chemiluminescence system.

### Cell viability assay

Cell viability was assessed using MTT assay. The cell lines were seeded in 96 well plates and allowed to grow for 1, 2, 3, 4 and 5 days. For drug susceptibility assay, cells were treated with various concentrations of 5-FU, DDP, L-OHP and DOC for 48 hours. Then 20 μl MTT (5mg/ml) was added to each well and incubated at 37°C for 4 hours. The medium was removed and formazan crystals were dissolved in DMSO. The optical density (OD) was measured at 490 nm using a microplate reader.

### Cell cycle analysis

The effect of RARα knockdown on cell cycle progression was determined by flow cytometry. The thymidine-synchronized cells were collected after G1 block. Then the cells were digested and centrifuged at 800 rpm for 10 min. After fixation in 70% ethanol at 4°C overnight, the cells were stained with PI solution (50 μg/ml PI and 100μg/ml RNase A in PBS) for detection.

### Colony formation assay

Cells were seeded at a density of 500 cells per well in six well plates and incubated for 14 days to form colonies. The cells were washed with PBS and stained with crystal violet. The number of colonies was counted using an inverse microscope. The experiments were carried out at least three times.

### Tumor xenografts

BGC-823 stablely transfected with vectors downregulating RARα or the control vectors were subcutaneously injected into the right oxter of 6 weeks old male BALB/c athymic nude mice for 18 days. Tumor volumes and weights were measured as previously described [28]. For drug susceptibility experiment, 5-FU (20 mg/kg) was administrated via intraperitoneally injection when tumor volumes were about 50 mm^3^. At the end of the 2 week dosing schedule, the mice were sacrificed with CO^2^ inhalation. Ethical approval was obtained from the Animal Care and Use Committee of the First Affiliated Hospital of Xiamen University before the beginning of experiments.

### Cell migration and invasion assay

Cell migration and invasion assay were detected by transwell assays. Cells were seeded in the upper chambers in serum-free media with or without the Matrigel membrane for 48 hour. Then cells migrating through the membrane were counted after they were stained with crystal violet.

### Statistical analysis

SPSS16.0 statistical software package (SPSS Inc., Chicago, IL, USA) was used to analyse statistical date. Data were expressed as mean ± SEM from at least three independent experiments. Statistics was evaluated using Student's t test or ANOVA analysis. IHC on tissue microarrays was analyzed by Pearson's χ^2^ test, and the survival rate was calculated by the Kaplan-Meier method. The data were regarded as significant when the *P* value was less than 0.05.

## SUPPLEMENTARY MATERIALS FIGURES


