# Correction: Positive feedback loop of IL-1β/Akt/RARα/Akt signaling mediates oncogenic property of RARα in gastric carcinoma

**DOI:** 10.18632/oncotarget.28906

**Published:** 2026-08-19

**Authors:** Hong-Yue Ren, Fan Liu, Gui-Li Huang, Yu Liu, Jin-Xing Shen, Pan Zhou, Wen-Ming Liu, Dong-Yan Shen

**Affiliations:** ^1^Department of Pathology, The Affiliated Southeast Hospital of Xiamen University, Zhangzhou 363000, Fujian Province, China; ^2^Department of Medical College, Xiamen University, Xiamen 361005, Fujian Province, China; ^3^State Key Laboratory of Cellular Stress Biology, Key Laboratory of the Ministry of Education for Coastal and Wetland Ecosystems, Department of Life Sciences, Xiamen University, Xiamen 361005, Fujian Province, China; ^4^Department of Biobank, The First Affiliated Hospital of Xiamen University, Xiamen 361003, Fujian Province, China; ^5^Department of Gastroenterology, Zhongshan Hospital, Gastroenterology Institute of Xiamen University, Gastroenterology Center of Xiamen, Xiamen 361003, Fujian Province, China; ^*^These authors have contributed equally to this work

**This article has been corrected:** Oncotarget analysis has confirmed a duplication of the Akt western blot found in Figures 5B and 6B. The authors have acknowledged this issue, stating that the Akt blot image for Figure 5B was inadvertently used twice. To resolve this, the authors provided the raw, uncropped Akt blot from the original experiments for Figure 6B. They have prepared a corrected version of Figure 6B using the appropriate image. The authors have stated that this correction does not alter the original results or conclusions of the paper and apologize for any inconvenience.

Original article: Oncotarget. 2017; 8:6718–6729. 6718-6729. https://doi.org/10.18632/oncotarget.14267

**Figure 6:**
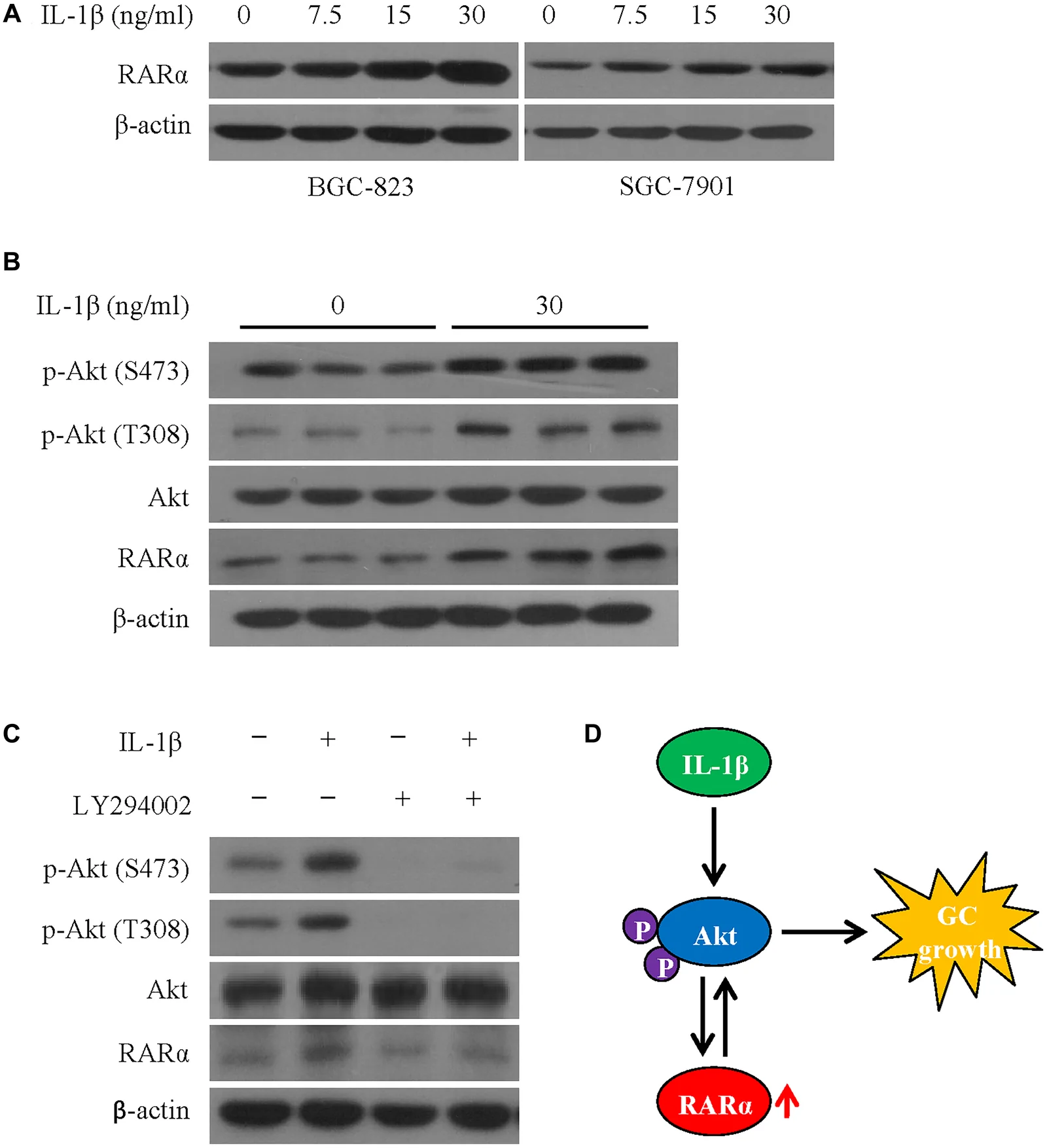
IL-1β induced RARα overexpression via activation Akt signaling. (**A**) Western blot analysis for RARα expression after treatment with different concentration of IL-1β for 24 hour. (**B**) Western blot analysis for RARα expression and the activation of Akt signaling after treatment with IL-1β for 24 hour in BGC-823. (**C**) After treatment with IL-1β and (or) LY294002 for 24 hour, expression of RARα, p-Akt and Akt were detected by Western blot in BGC-823. IL-1β, 30 ng/ml; LY294002, 1 μM. (**D**) Schematic diagram for the positive feedback loop of IL-1β/Akt/RARα/Akt signaling.

