# Supplementary material for: Positive feedback loop of IL-1β/Akt/RARα/Akt signaling mediates oncogenic property of RARα in gastric carcinoma
Source: Oncotarget. 2016 Dec 27;8(4):6718–29. doi: 10.18632/oncotarget.14267 (PMC5351665; doi:10.18632/oncotarget.14267)
Supplement: Supplementary file 1 [file oncotarget-08-6718-s001.pdf]

Positive feedback loop of IL-1β/Akt/RARα/Akt signaling mediates oncogenic property of RARα in gastric carcinoma

SUPPLEMENTARY FIGURES

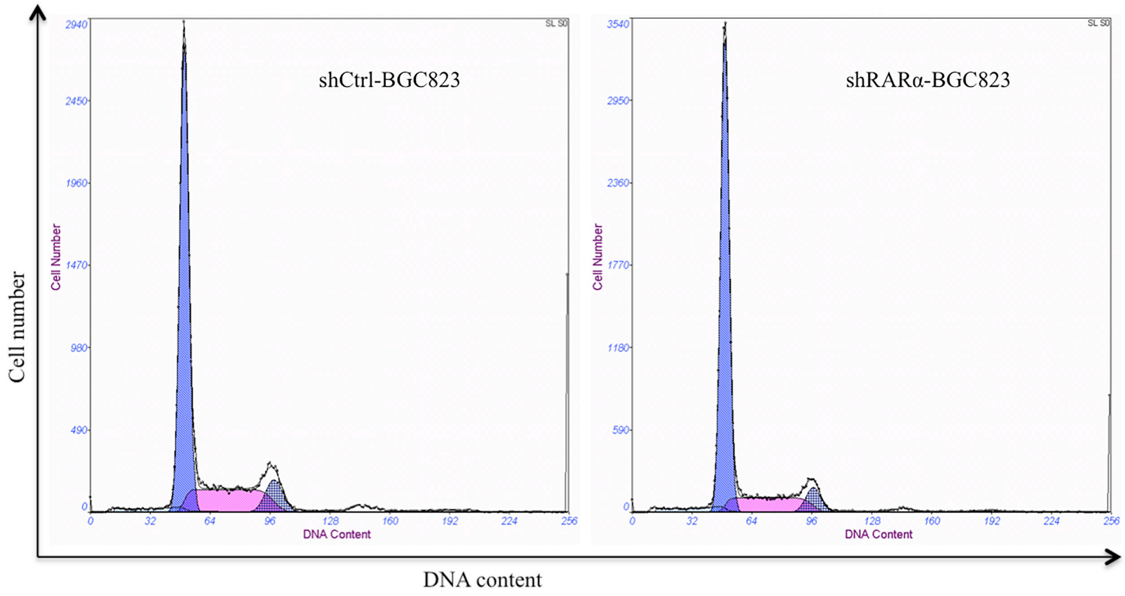

Supplementary Figure 1: The role of RARα in GC cell cycle. Cell cycle was assessed by flow cytometry after RARα knockdown.

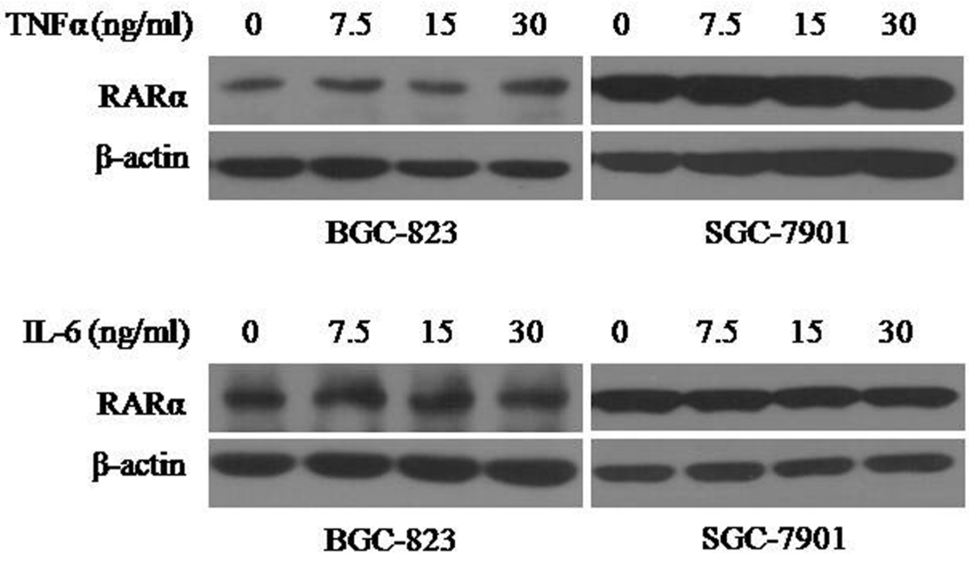

Supplementary Figure 2: The effect of TNFα and IL-6 on RARα expression. Western blot analysis for RARα expression after treatment with different concentration of TNFα and IL-6 for 24 hour.
